# Paper-based aptamer-antibody biosensor for gluten detection in a deep eutectic solvent (DES)

**DOI:** 10.1007/s00216-021-03653-5

**Published:** 2021-10-06

**Authors:** Rossella Svigelj, Nicolò Dossi, Cristian Grazioli, Rosanna Toniolo

**Affiliations:** Department of Agrifood, Environmental and Animal Science, University of Udine, via Cotonificio 108, 33100 Udine, Italy

**Keywords:** Paper-based biosensor, Electrochemical detection, Deep eutectic solvents, Aptamers, Gluten

## Abstract

Paper has been widely employed as cheap material for the development of a great number of sensors such as pregnancy tests, strips to measure blood sugar, and COVID-19 rapid tests. The need for new low-cost analytical devices is growing, and consequently the use of these platforms will be extended to different assays, both for the final consumer and within laboratories. This work describes a paper-based electrochemical sensing platform that uses a paper disc conveniently modified with recognition molecules and a screen-printed carbon electrode (SPCE) to achieve the detection of gluten in a deep eutectic solvent (DES). This is the first method coupling a paper biosensor based on aptamers and antibodies with the DES ethaline. Ethaline proved to be an excellent extraction medium allowing the determination of very low gluten concentrations. The biosensor is appropriate for the determination of gluten with a limit of detection (LOD) of 0.2 mg L^−1^ of sample; it can detect gluten extracted in DES with a dynamic range between 0.2 and 20 mg L^−1^ and an intra-assay coefficient of 10.69%. This approach can be of great interest for highly gluten-sensitive people, who suffer from ingestion of gluten quantities well below the legal limit, which is 20 parts per million in foods labeled gluten-free and for which highly sensitive devices are essential.

**Graphical abstract:**

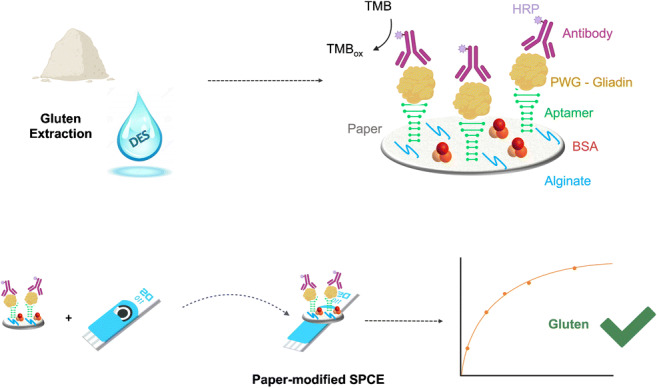

## Introduction

Paper displays interesting physical and physicochemical properties, such as adsorption properties, capillary action, and high surface-to-volume ratio, and allows immobilization of biomolecules [1]. It has been applied in many different research fields, such as in the development of sensors, microfluidic devices, and point-of-care(POC) diagnostic tools [2]. In recent decades, POC tests based on paper have been developed for glucose and other important bioactive molecules [3, 4]. Currently, paper continues to be employed as material for the production of widely used sensors such as pregnancy tests, strips to measure blood sugar, and COVID-19 rapid tests [5, 6].

Besides paper strips, patterned paper has also been used as a platform for the implementation of portable, low-cost bioassays aimed at use in developing countries [7, 8]. In addition, electrochemical detection for paper-based microfluidics was also proposed for the determination of low levels of analytes in biological samples and complex sample matrixes [9].

The need for new low-cost analytical devices is growing, and the use of these platforms will be extended to different assays both for the final consumer and within laboratories [10, 11]. Among the most relevant points in the use of this material, there are advantages such as biocompatibility and biodegradability, low cost, and ease of production [12]. These aspects have led to a growing interest in the development of paper-based analytical devices (PADs), such as smart labels [13], gas sensors [14, 15], and sensors combining electrochemical and visual readouts [16]. PADs have successfully found application in diagnostics [4], environmental monitoring [17], and food control [18].

To date, paper-based gluten sensors such as lateral flow devices are commercially available, indicating the presence or absence of gluten, with a limit of detection (LOD) of around 4 mg L^−1^. They can be used for potentially contaminated surfaces and to check for gluten contamination of raw or processed materials [19], but they are not suitable for sensitive gluten quantification.

As is well known, celiac disease is triggered by the ingestion of gluten in people predisposed to the disease [20]. In the future, it will be increasingly necessary for consumers to monitor food directly at home. Thus, the development of low-cost platforms that are easy to use and highly sensitive is of growing interest [18].

Gluten is composed of a complex mixture of water-insoluble storage proteins; among them, gliadin is commonly used as the analytical target to quantify gluten in food. The most commonly used solvent in gluten quantification methods is a 60% (*v*/v) ethanol-water solution; however, this method is not able to completely extract gluten from processed food [21]. Reducing and disaggregating agents have also been used in combination with alcohol solutions to overcome this problem [22, 23]. Nevertheless, both 2-mercaptoethanol and denaturants used in the extraction cocktails can interfere in the subsequent protein recognition, affecting the quantification results [24]. Thus, substantial sample dilutions are usually needed. The problem regarding the complete extraction of gluten proteins from food makes the determination of gluten a continuing challenge and an open topic in which research advances are needed [25].

Recently, an alternative method of extraction using a deep eutectic solvent (DES) was proposed [26]. This approach allows the direct measurement of the extracted sample in the DES ethaline (choline chloride:ethylene glycol, 1:2), exploiting the biocompatibility of the eutectic solvent with molecules such as DNA and antibodies.

DESs are formed thanks to the interaction between a hydrogen bond donor (HBD) and a hydrogen bond acceptor (HBA) [27]. They present low vapor pressure and a high ability to dissolve molecules of different nature; they are green, easy to produce, and low-cost [28, 29]. For these reasons, the use of DESs is expanding in different fields [30–33], in fact, in recent years, they have been applied in the extraction of various molecules [34–36] and in different research areas including organic synthesis, electrochemistry, and biocatalysis [37–39].

Here we describe a paper-based electrochemical sensing platform that uses a paper disc conveniently modified with recognition molecules and a screen-printed electrode to achieve the detection of gluten in DES. This analytical platform allows the use of a single screen-printed carbon electrode (SPCE) for the analysis of several paper discs. This provides the possibility for reduced use of SPCEs and a considerable decrease in the cost of each measurement.

This sandwich-type electrochemical biosensor exploits an aptamer and antibody pair for the detection of gluten. Aptamer-antibody sandwich assays show enhanced sensitivity and specificity [40, 41]. For this reason, a Gli4-T aptamer was employed as the capturing element, while a 401/21 antibody was used as detection probe. This platform was designed to provide a low-cost and sensitive tool for the fraction of highly gluten-sensitive people, who suffer from ingestion of gluten quantities well below the legal limit which is 20 parts per million in foods labeled gluten-free and for which highly sensitive devices are essential [42].

## Materials and methods

### Chemicals and reagents

All chemicals used were of analytical reagent grade and were employed without further purification. HPLC-purified 5′-tagged (-NH_2_) aptamers were obtained from Tema Ricerca (Bologna, Italy). The Gli4 complete aptamer sequence and the truncated sequence are shown in Table 1. The 401/21 antibody and enzyme-linked immunosorbent assay (ELISA) kit were supplied by Bio-Check (UK). Gliadin standard was kindly provided by the Prolamin Working Group (PWG). Ethaline was supplied by Scionix Ltd. (London, UK) and employed as received (Table 2). Salts for buffer solutions, bovine serum albumin (BSA), 1 M Tris/HCl pH 7.4, phosphate-buffered saline (PBS) 10×, sodium alginate, 1-ethyl-3-(3-dimethylaminopropyl)carbodiimide (EDC), *N*-hydroxysuccinimide (NHS), and a 3,3′,5,5′-tetramethylbenzidine (TMB) liquid substrate system for ELISA solution were obtained from Sigma-Aldrich (Italy). With regard to filter paper, 50 cm × 50 cm foils, 67 g/m^2^, 0.13 mm thick, were purchased from Labor (Cordenons, Italy) and 0.18 mm-thick paper from Whatman (Maidstone, UK).

**Table 1:** Nucleotide sequences of the aptamers

Name	Sequence	Length	∆G (kcal/mol)
Gli4	CCA GTC TCC CGT TTA CCG CGC CTA CAC ATG TCT GAA TGC C	40 bases	−1.72
Gli4-T	CTA CAC ATG TCT GAA TGC C	19 bases	−2.86

**Table 2:** Formula, component molar ratio, and viscosity of DES adopted

DES	Salt-HBA	HBD	Molar ratio	Viscosity (25 °C) mPa s	Viscosity (55 °C) mPa s
Ethaline			1:2	37	24

High-purity deionized water (purified by an Elgastat UHQ PS system, Elga, High Wycombe, UK) was used for all washing operations.

### Paper-based biosensor assembly

**Fig. 1:**
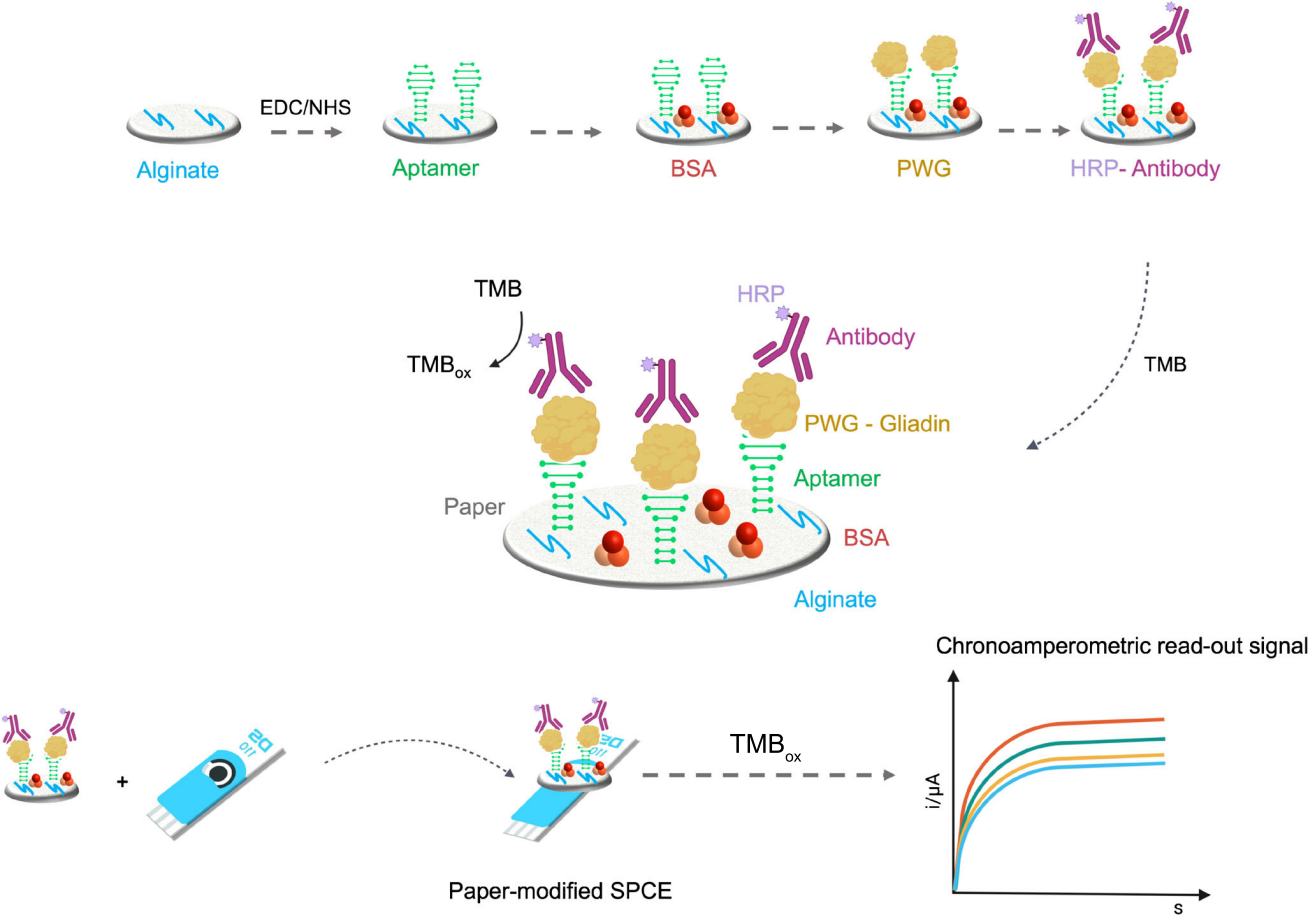
Schematic representation of the gluten paper-based biosensor design and working principle

Filter paper was cut into discs of 6 mm. As shown in Fig. 1, discs were soaked in 10 μL of sodium alginate (0.05% *w*/*v*) at 4 °C overnight. After a washing step, the polysaccharide-modified paper was ready for use. Then, 10 μL of a mixed solution (EDC 0.4 M and NHS 0.2 M) was used to activate the carboxylic groups of the polysaccharide. The 5′ amined aptamer (1 μM) was covalently immobilized on the paper during 2 h incubation, and then the surface was blocked for 15 min with a solution of 1% BSA. Subsequently, solutions of increasing concentration (between 0.1 and 10 mg L^−1^) of PWG gliadin in buffer (Tris 50 mM, NaCl 250 mM, MgCl_2_ 5 mM) or alternatively in ethaline were incubated for 20 min. Next, a solution of the anti-gluten antibody was incubated for 10 min. After each step, discs were washed with buffer solution. Finally, electrochemical transduction was performed by adding 40 μL of TMB solution, and after 3 min of the enzymatic reaction the paper disc was transferred onto the SPCE and measured by chronoamperometry at 0 V (Figure1).

### Electrochemical measurements

All electrochemical measurements were performed with a µAutolabIII/FRA2 potentiostat controlled by Nova 2.1 software (EcoChemie, the Netherlands). Disposable screen-printed carbon electrodes (SPCE) ref. C110 were purchased from Metrohm DropSens (Metrohm Italiana s.r.l., Varese, Italia).

### Sample preparation and extraction procedure

To prevent contamination, samples were prepared in a laboratory separated from where analyses were performed. Gluten was extracted with ethaline from corn flakes, and a gluten-free flour purchased from a local supermarket.

A 0.1 g sample of the obtained powder was extracted in vials with 1 mL of pure DES [43]. Vials were shaken in a vortex for 2 min, and then they were left at 55 °C for 45 min. Next, samples were shaken again for 2 min and centrifuged for 10 min at 5000 rpm. Depending on the level of gluten, the supernatant was applied directly on the electrode surface or properly diluted in 100% DES before the analysis.

## Results and discussion

### Modification of SPCEs with paper

**Fig. 2:**
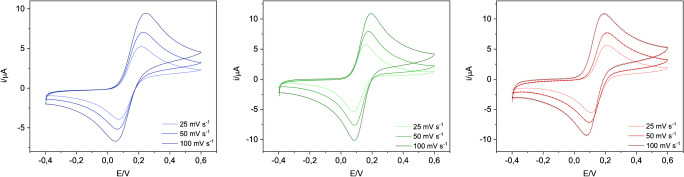
Cyclic voltammograms of bare SPCE (blue), filter paper-modified SPCE (green) and Whatman paper-modified SPCE (red) recorded at different scan rates (25, 50, 100 mV/s) using the redox probe [Fe(CN)_6_]^4−^ 2 mM in PBS buffer and KCl 3 mM

Initially, the modification of the SPCE with the paper disc was evaluated with cyclic voltammetry at different scan rates (25, 50, 100 mV/s) using the redox probe ferrocyanide 2 mM in PBS buffer and KCl 3 mM as supporting electrolyte. Cyclic voltammograms recorded at the bare SPCE and filter paper-modified SPCE and Whatman paper-modified SPCE are presented in Fig. 2. A well-defined and quasi-reversible redox process was found for the redox couple [Fe(CN)_6_]^4−^/ [Fe(CN)_6_]^3−^ for all the setups, with a peak-to-peak separation (ΔE_p_) recorded at 50 mV/s of 152 mV on bare SPCE, 91 mV on filter paper, and 110 mV on Whatman paper. These results show that the electron transfer on paper-modified SPCE is very similar to that recorded at bare SPCE, suggesting that good electrolytic contact was achieved. However, the measurements performed on Whatman paper showed a background noise at high scanning speeds.

### Gliadin quantification by paper-based biosensor

**Fig. 3:**
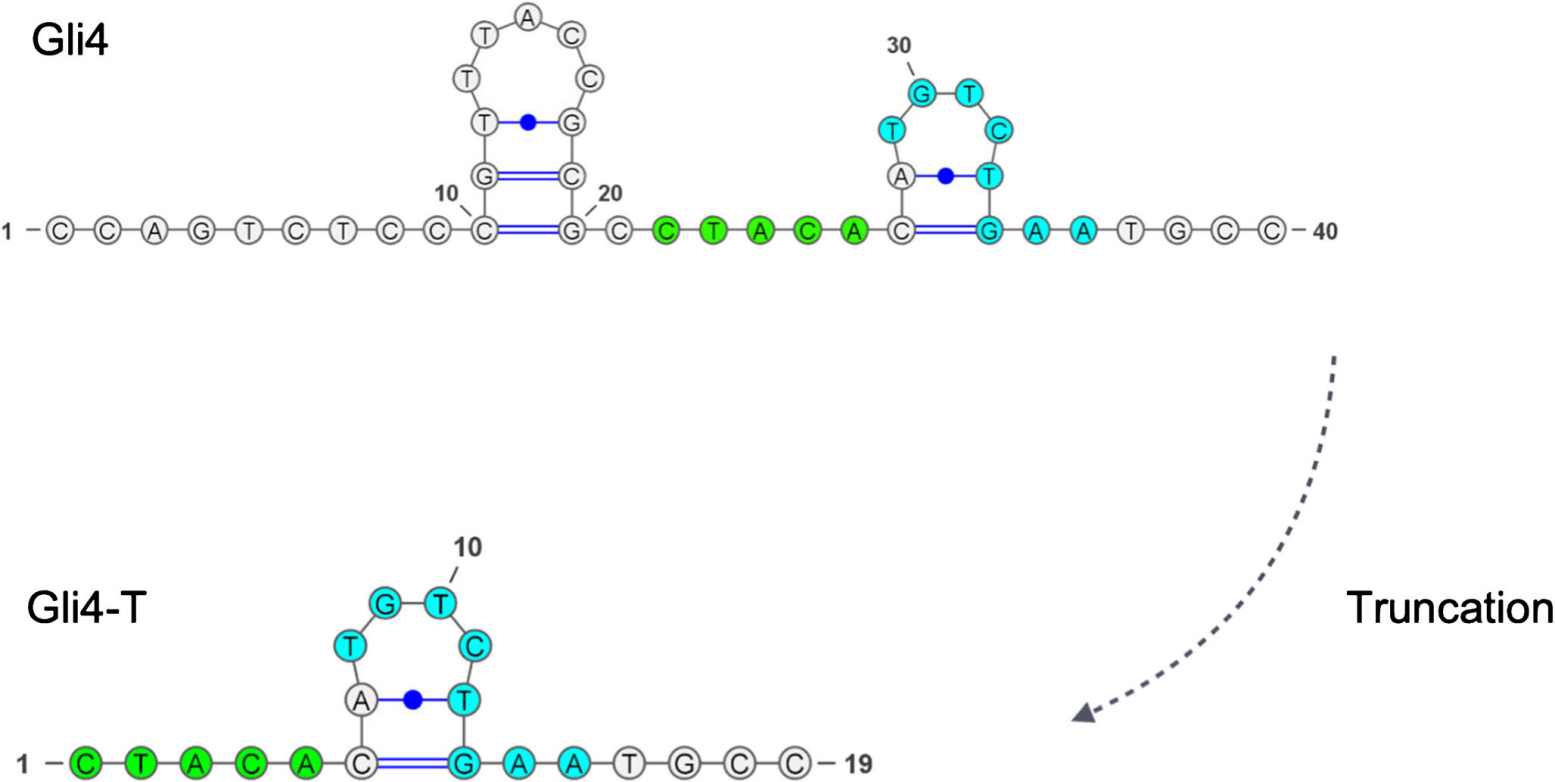
Secondary structures of Gli4 and truncated aptamer Gli4-T. Motifs responsible for the binding with gliadin are highlighted

The principle of the biosensor is illustrated in Fig. 1. In this approach, the truncated aptamer Gli4-T was employed as capture element while the 401/21 antibody was employed as the signaling element. In a previous work, the process of truncation of non-essential nucleotides was reported to improve the accessibility of the target to the aptamer, allowing the formation of a stronger aptamer-target complex [44]. Gli4-T is the truncated aptamer derived from Gli4 which preserves a motif capable of effectively binding gliadin (Fig. 3) [45, 46]. Gli4-T has a dissociation constant (K_d_) of 148 ± 8 nM in aqueous buffer, and a K_d_ of 515 ± 144 nM in ethaline [43].

**Fig. 4:**
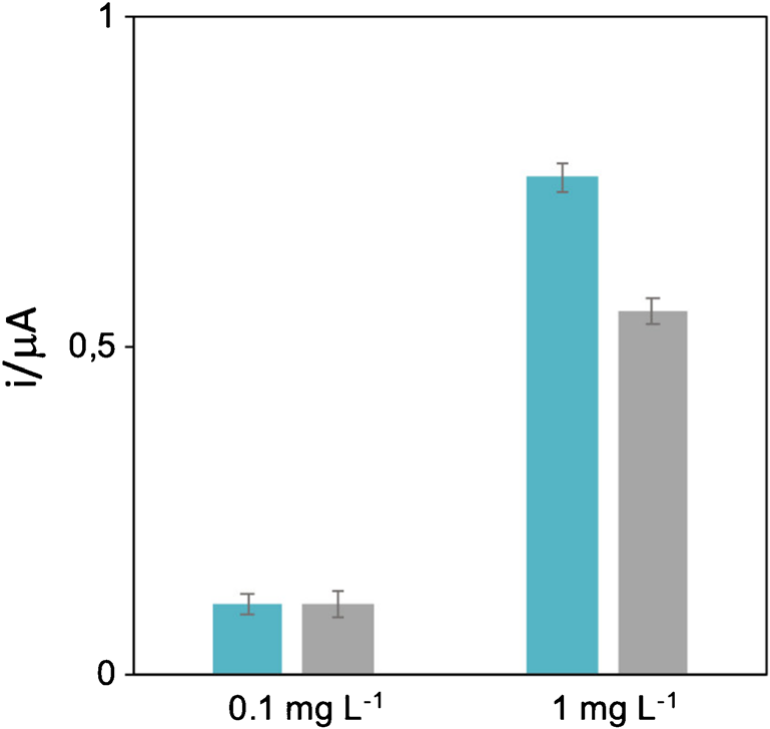
Comparison of the performance of two different papers (laboratory filter paper in blue, Whatman paper in gray) at 0.1 mg L^−1^ and 1 mg L^−1^ of PWG-gliadin

The paper-based sensor was designed using common laboratory filter paper cut into discs of 6 mm. A polysaccharide with multiple functional groups was employed as surface modifier for paper on which the aptamer was covalently immobilized [47]. Sodium alginate was dropped onto the paper, and the carboxyl groups were then activated by EDC/NHS and subsequently the amino-modified aptamer was immobilized. After blocking the surface with BSA, PWG-gliadin solutions were tested in the range of 0.1–10 mg L^−1^. Lastly, the antibody labeled with horseradish peroxidase (HRP) enzyme was incubated and TMB substrate added. The enzyme catalyzed the oxidation of TMB in the presence of hydrogen peroxide, then the chronoamperometric detection was performed at 0 V. Both filter paper and Whatman paper were tested in the development of the assay. As can be observed in Fig. 4, at lower concentrations of analyte there was little difference in the performance, but at higher concentrations of gliadin (1 mg L^−1^), the common filter paper showed a higher signal ratio, proving to be the best option for the assay development.

**Fig. 5:**
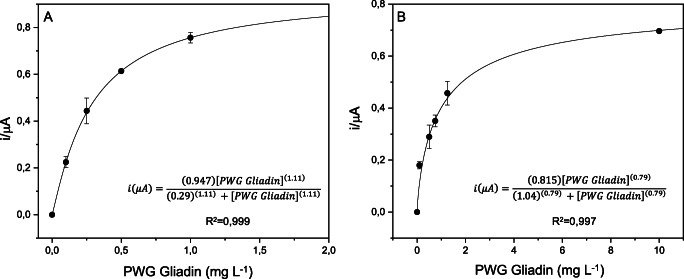
Calibration curves of the sandwich assay in aqueous in buffer (**A**) and ethaline (**B**) fitted to the Hill function

For the sake of comparison, calibration curves were conducted both in aqueous buffer and in ethaline. As can be seen in Fig. 5, both calibration curves fitted to the Hill function, with a correlation of 0.999 in aqueous buffer and 0.997 in ethaline.

The calibration in DES allowed to expand the measurement to 10 mg L^−1^, while the calibration in aqueous buffer already reached the saturation at a concentration of 2 mg L^−1^ of gliadin. The response of the biosensor was determined in a range from 0.1 to 1 mg L^−1^ of gliadin in aqueous buffer and from 0.1 to 10 mg L^−1^ in ethaline.

The reproducibility of the method was very good; the calculated intra-assay CV % in aqueous buffer was 6.64 considering all tested concentrations on three different days, while in ethaline it was 10.69%.

The results obtained with this sandwich sensor proved to be interesting for the sensitive, low-cost detection of gluten. In addition, the obtained LOD, calculated as three times the standard deviation of the blank signal divided by the slope calculated for the linear dynamic range, is 0.2 mg L^−1^ of gluten, which successfully competes with the assays described in the literature and on the market, as can be seen in Table 3.

**Table 3:** Comparison of the analytical performance of different approaches for gluten detection

Method	Recognition element	Format	Detection range (gluten)	LOD (gluten)	Ref.
EIS	Aptamer	Direct (label-free)	10–100 mg L^−1^ and 100–2000 mg L^−1^	5 mg L^−1^	[48]
EIS	Antibody	Direct (label-free)	10–40 mg L^−1^	10 mg L^−1^	[49]
ELISA (RIDASCREEN^®^ Gliadin, R-Biopharm)	R5 mAb	Sandwich	0.01–0.16 mg L^−1^	1 mg L^−1^	[50, 51]
Lateral flow assay (RIDA^®^QUICK Gliadin, R-Biopharm)	R5 mAb	Immunochromatographic	–	4.4 mg L^−1^	–
Paper-based aptamer-antibody sandwich	Aptamer-antibody	Sandwich	0.2–20 mg L^−1^	0.2 mg L^−1^	This work

### Application of the assay to food samples extracted in DES

The paper-based biosensor here developed can be applied in the determination of gluten in gluten-free foods to provide more information and safety to celiac people. To demonstrate its applicability to gluten quantification, two real food samples were analyzed. We tested a gluten-free flour sample and a corn flakes sample using ethaline for the extraction. Accordingly, we also performed the quantification with an ELISA, and the results are compared in Table 4.

**Table 4:** Comparison between the results obtained with the paper-based biosensor and the ELISA

Sample	Paper biosensor (mg L^−1^)	ELISA (mg L^−1^)
Gluten-free flour	2.68 ± 0.01	2.3 ± 0.1
Corn Flakes	21.33 ± 0.08	20.50 ± 0.09

The quantification of gluten in the flour sample with the paper-based method gave a gluten content of 2.68 ± 0.01 mg L^−1^, a result comparable to the one obtained with the classic ELISA (2.3 ± 0.1 mg L^−1^) of gluten. Moreover, the sensor also worked well for the corn flakes sample with a higher gluten content (21.33 ± 0.08 mg L^−1^) in good agreement with the results obtained from the ELISA test (20.50 ± 0.09 mg L^−1^). These results proved that this method can be successfully used for the reliable quantification of gluten in foods labeled as gluten-free and can be a valid alternative to current methods. Moreover, to ensure the effectiveness and applicability of the method, we evaluated the possible interference caused by other proteins. For this purpose, chickpea flour with high protein content was used. After extraction with ethaline, the sample was tested with the paper-based sensor. This naturally gluten-free sample gave comparable signals as blanks, as can be seen in Table 5, demonstrating that this method shows no interference from the presence of other proteins in the sample.

**Table 5:** Selectivity assay results comparing blanks signals with chickpea flour

Sample	Signal (μA)
Blanks	0.45 ± 0.01
Chickpea Flour	0.45 ± 0.02

## Conclusions

In this work we have designed a simple, low-cost, and easy-to-use biosensor. This is the first method coupling a paper biosensor based on aptamers and antibodies with a DES. We coupled a paper disc with recognition biomolecules for the electrochemical determination of gluten extracted in DES, providing a cheap detection device in comparison with traditional methods. This approach could enable a substantial advance in the analysis of gluten, being very economical and sensitive at the same time. Very important aspects to take into consideration are the use of biodegradable materials such as paper and DES and the advantage of using a single SPCE for a whole set of measures. Moreover, this biosensor has proven to be appropriate for the determination of very low concentrations of gluten, with a LOD of 0.2 mg L^−1^ of sample. The ELISA R5 Mendez method remains the only method recommended by the Codex for gluten analysis; however, all advances in this area can bring important improvements in the quantification of gluten. This approach can be of great interest for highly gluten-sensitive people who suffer from ingestion of gluten quantities well below the legal limit and for whom highly sensitive devices are essential.

## Data Availability

Not applicable.
